# Supplementary material for: Short-Term Efficacy and Safety of Cataract Surgery Combined with Iris-Fixated Phakic Intraocular Lens Explantation: A Multicentre Study
Source: J Clin Med. 2021 Aug 19;10(16):3672. doi: 10.3390/jcm10163672 (PMC8397074; doi:10.3390/jcm10163672)
Supplement: Supplementary file 1 [file jcm-10-03672-s001.zip › jcm-1292844-supplementary.pdf]

Supplemental Table S1. Intraocular lenses implanted during cataract surgery

IOL	No.
Lentis Mplus (Teleon Surgical, Spankeren, Netherlands)	17
ATLisa (Carl Zeiss Meditec, Jena, Germany)	16
MN60MA (Alcon, Geneva, Switzerland)	11
Lentis Mplus Toric (Teleon Surgical, Spankeren, Netherlands)	9
XY1 (HOYA, Tokyo, Japan)	6
ZCB00 (AMO Japan, Tokyo, Japan)	5
ZCB00V (AMO Japan, Tokyo, Japan)	5
MA60MA (Alcon, Geneva, Switzerland)	3
Acriva Trinova Toric (VSY Biotechnology, Leinfelden-Echterdingen, Germany)	2
ALSAFIT (Alsanza Medizintechnik und Pharma GmbH, Pfullingen, Germany)	2
ATLisa Toric (Carl Zeiss Meditec, Jena, Germany)	3
Lentis Mplus Toric MF20 (Teleon Surgical, Spankeren, Netherlands)	2
Lentis MplusX (Teleon Surgical, Spankeren, Netherlands)	2
SND1T4 (Alcon, Geneva, Switzerland)	2
ZCV300 (AMO Japan, Tokyo, Japan)	2
FineVision (PhysIOL, Liège, Belgium)	1
Lentis MplusX Toric (Teleon Surgical, Spankeren, Netherlands)	1
MN60AC (Alcon, Geneva, Switzerland)	1
PCB00V (AMO Japan, Tokyo, Japan)	1
RayOne (Rayner, West Sussex, United Kingdom)	1
SN60WF (Alcon, Geneva, Switzerland)	1
SN6AT5 (Alcon, Geneva, Switzerland)	1
ZCV150 (AMO Japan, Tokyo, Japan)	1
ZMB00 (Alcon, Geneva, Switzerland)	1

IOL, intraocular lens.

Supplemental Figure S1. Comparison of subjective astigmatism between the pIOL materials.

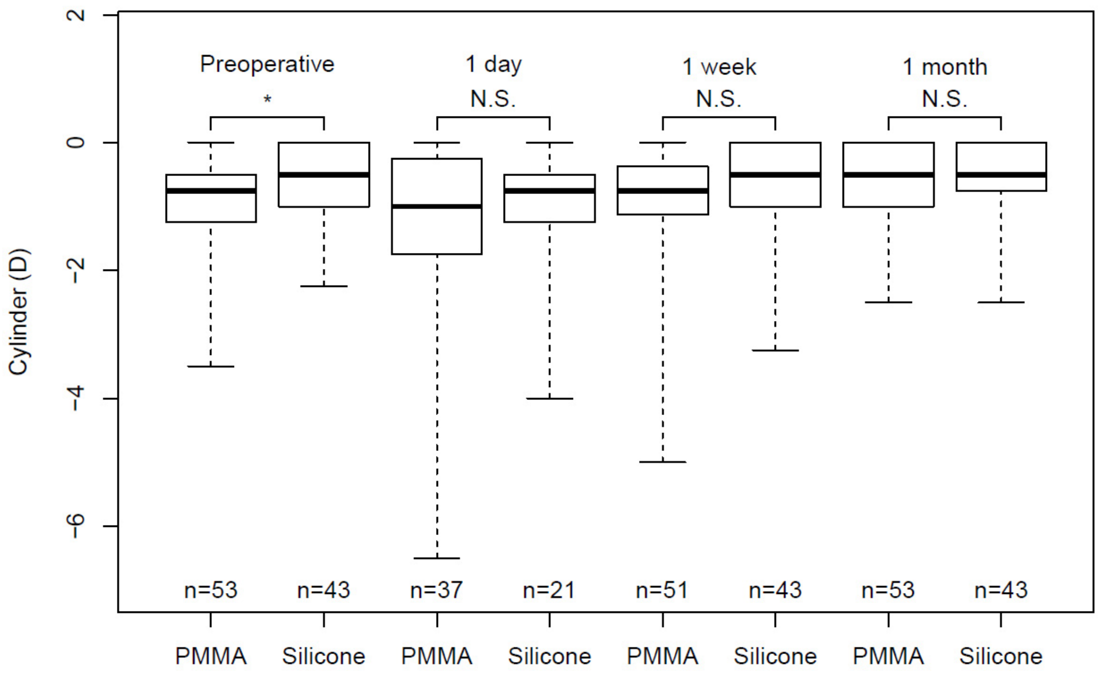

* indicates a significant difference between baseline and each time point. N.S., not significant; D, diopters; PMMA, polymethyl methacrylate; pIOL, phakic intraocular lens.
